# Investigating the Effectiveness of Recycled Agricultural and Cement Manufacturing Waste Materials Used in Oil Sorption

**DOI:** 10.3390/ma15010218

**Published:** 2021-12-28

**Authors:** Marina Valentukeviciene, Ramune Zurauskiene

**Affiliations:** Environmental Engineering Faculty, Vilnius Gediminas Technical University, 10223 Vilnius, Lithuania; ramune.zurauskiene@vilniustech.lt

**Keywords:** recycled materials, environmental engineering, water reuse, oil removal

## Abstract

This research investigates how sorbents made from recycled waste materials affect the properties of water used to remove residues flushed from oil tanks transported by rail. The mineral sorbent was added to water following the flushing process. Water temperatures were maintained at 21 °C and 70 °C for a contact period of 30 min. The experiments demonstrated that: when the sorbent is active, turbidity removal efficiency was about 64%; color removal efficiency of 56% was obtained; and total iron concentration removal was approximately 68%. The effect of the characteristics of the materials on the adsorption capacity was evaluated using the removed amount of oil per one gram of every sorbent. It was found that straw sorbent oil adsorption capacity was up to 33 mg/g, peat sorbent 37 mg/g, and mineral sorbent 1.83 mg/g. The following were also measured during the experiment: temperature, pH, chemical oxygen usage, total iron concentrations, suspended matter, and oil concentrations. The findings show that recycled sorbents obtained from waste materials are environmentally sustainable and can be reused to treat water that has been used to flush oil transported in rail tanks.

## 1. Introduction

The most significant sources of oil pollution include those produced by factories, combustion engines, and/or leaking vehicle parts [1]. The most common pollutants are gasoline, lead, zinc, chrome, iron, arsenic, pesticides, nitrates, and toxic compounds [2].

Typically, oil residue is cleaned from rail tanks that have been used to transport fluids, although the process of removal can be highly problematic [3]. This process comprises the following steps. Firstly, emptying the tank by removing carbohydrate until the level of the oil product is sufficiently lowered so that cleaning equipment can be used.

Secondly, introducing air to flush out harmful gases, but only to the point that steam generated by the oil product does not exceed the highest allowed concentration. Then, the third step is to use special cleaning equipment to cleanse the tank wall and floor bed. This is achieved through oil flow created by the cleaning equipment used to measure both the concentration of carbohydrates in the gasses and static electricity energy. Note that both of these procedures should be performed at the same time. Finally, there are a few more steps: flushing the dissolved oil particles and dispersive sludge into a special container or via a pipeline; flushing the emulsified water from the tank; conducting sorption during degassing, forced ventilation and introduction of air; by the end, controlling the quality of the sludge that has been removed.

Currently, several methods are used to remove sludge from the bed of the tank floor. One of these is the hydraulic method, which is energy efficient and economically efficient when compared to other hydraulic methods used to clean tanks. In addition, the absence of complicated parts in this form of cleaning makes for a highly reliable system which requires little oversight, thereby saving time [3]. Sludge needs to be reduced during the above mentioned procedures, although this usually extends the time between cleaning, and increases the amount of wastewater in the capture vessel, as well as the oil product transportation systems [3].

Cleaning technologies used to purify water include reverse osmosis with nano-filtration and ultrafiltration, ultrafiltration with adsorption by activated charcoal, electrochemical oxidation, biological treatment, and flocculation [4]. These are discussed in numerous published articles that review various transport cleaning wastewater management methods used throughout the world [5]. Such methods include membrane filtration, adsorption, floatation and chemical coagulation, cleaning using dispersion resulting from ultrasound, nano-technologies, catalysis processes, and other hybrid technologies.

Electrochemical methods can be used to destabilize oil product emulsion in wastewater, and the most commonly used electrochemical methods for cleaning are electrocoagulation and enhanced flocculation [5]. Hydrocarbons and suspended materials can be removed from wastewater by a physical-chemical method using coagulants and flocculants. During the coagulation process various particles (suspended, colloidal particles, oil) are destabilized causing them to attach to other particles. With this method, ever-larger particle flakes form, so that when flake density becomes higher than that of water, they sink to the bottom of the tank and are removed using the sedimentation method [5]. The aforementioned authors [6] compared membrane bioreactor processes (MBP) with chemical coagulation and ozonation, and concluded that better results could be achieved using MBP instead of coagulation.

When sorption is used to treat wastewater, oil products are removed using an adsorbent, for example: polypropylene, activated carbon, polyacrylamide, the base of which is made of chitosan [5]. Of all the methods used to remove pollution produced by wastewater treatment technologies, sorption proved to be the best due to its low price, simplicity, and effectiveness [7].

Oil products in wastewater take different forms, for example unstable, dispersed oil products as well as stable emulsified oil products that float on the surface [8]. A number of researchers [9] have demonstrated that electrocoagulation using iron and aluminum electrodes remove oil products from rail tanks. Their experiments showed a reduction by 90.18% of Chemical Oxygen Demand indicators in the flushed wastewater. Other researchers [10] have investigated the use of natural fibers such as rice husks to treat water polluted with oil products. One clear advantage of this cleaning method is its ability to absorb different compounds from multicomponent solutions, thereby achieving a high level of reused water quality, especially from highly polluted sources. Notwithstanding, sorbent made from rice husks has limitations due to the prior removal of most remaining pollutants. This is due to the fact that the rice peel and straw contain high levels of silicon dioxide.

Skimming, precipitation, centrifugation, and biological methods are common techniques used to separate oil pollution from contaminated wastewater. Yet, most of these are unable to remove emulsified oil products, because the size of the compound particles is small. Moreover, proposed new technologies are expensive and do not effectively remove quantities of residual oil particles [11]. Since the membrane method is complicated, sorption methods are more acceptable when treating reused wastewater.

Previous research [11] shows that sorbents able to adsorb oil products can be divided into three main groups:

(1) Inorganic mineral products
(2) Organic synthetic products
(3) Organic natural products.

Accordingly, researchers have explored the removal of oil products from wastewater using barley straw, their aim being to evaluate the possibility of removing oil products using naturally disintegrating sorbents [11]. Various types of oil products were used in the experiments: sea Balaiem, Western Desert crude oil, Russian oil, and Arabian heavy crude oil. It was found that by increasing the temperature, sorption capacity rises, whereas at low temperatures oil particles tend to clog the pores of a sorbent, thereby preventing the absorption of oil. An attempt was made to remove adsorbed oil from the sorbent by mechanically pressing it several times and using it repeatedly for oil particle sorption. After three cycles, sawdust sorption capacity was measured which was equal to 50% from initial sorption capacity.

Thus, the main aim of our research was to identify the most effective, recyclable sorbents that can remove oil and other pollutants from reused wastewater obtained from the cleaning of railway tanks.

## 2. Materials and Methods

Storm-water that has been captured is commonly used to remove oil products residuals and other pollutants from railway transport tanks. The contaminated wastewater in the rail tank is eliminated from the washing area using special pumps and then stored in a reservoir. Oil, has a chemical composition of lipids, which, due to their structure, liquefy at higher temperatures and solidify at lower temperatures. During washing, hydraulic pumps inject the water into a heat exchange unit where it is heated to 70 °C. Hot water is then fed to the cleaning equipment, close to which are 14 cisterns, each with a capacity of 700 m^3^. Each of the cleaning devices has four steam supply stands, capable of processing four tanks at once. The inside of the tank is washed with the heated water, thereafter the wastewater is treated in a separator that removes the oil products which allows the treated wastewater to be reused.

The operator of the cleaning equipment first places the nozzle of the steaming device into the tank’s interior, then opens the steam valve to eject hot water at pressure. The tank is steamed for 20 min until the residue liquefies. Upon completion, the inner valve at the top of the rail tank is opened and steaming of the cistern resumes. This expels the oil products and voids the tank, with the result that the liquefied oil product residue drains into a collecting repository. Once the residue has been drained, the steam is turned off and the steaming equipment is withdrawn.

Water is fed into the nozzles of the washing apparatus impeller which spins on horizontal and vertical axles. For railway transport, most cleaning technologies are performed using high pressure and spinning nozzles, the cleaning being performed through an aperture in the center of the cistern top.

The contaminated wastewater from the cistern drains out through the pipelines to the primary treatment equipment. Sand and other sinking materials are separated in two sand and grease precipitators which have a depth of two meters. Oil products in the cleaning equipment rise to the surface due to the difference in density and are collected with the brush skimmer. Wastewater collected from the cleaning equipment is stored in reservoirs and re-used for cleaning processes. The optic method is used to identify if the wastewater is no longer recyclable due to its increased color and turbidity, in which case it is then returned for additional treatment.

Oily wastewater was obtained directly after washing processes were conducted with water at 70 °C. After standing still at the reservoirs, the temperature was lowered to 21 °C. All experiments were conducted with both temperatures to obtain the best reliable results.

Three different sorbents were used for experimental wastewater treatment:

- Gypsum granules, produced by absorbing granule limestone. According to the manufacturer’s specification it is recyclable sorbent for oil compounds removal
- Barley straw sorbent, obtained from local farmers as a waste product due to its low quality
- Hydrophobic peat sorbent, which can be used to absorb large amounts of oil products.

Two natural sorbents were used in this research (waste straw from a local Lithuanian farming area, and waste peat from an excavation area (JSC Durpeta)), and one mineral sorbent from recycled wasted materials produced by JSC Akmenes Cementas (Naujoji Akmene, Lithuania). All sorbents were dried in laboratory conditions with an approximate air humidity of 70%, and room temperature equal to 18 °C, before being mixed with wastewater. Natural sorbents were mechanically cut and sieved into useful fractions with the size varying in a range of approximately 2.0–3.0 cm. Mineral sorbent was obtained directly from the producing factory in fractions approximately between 4–6 mm. The straw sorbent has an approximate bulk density—0.04 g/cm^3^, peat sorbent has an approximate bulk density of 0.03 g/ cm^3^ and the highest density was measured for the mineral sorbent, equal to 0.75 g/ cm^3^ from cement production recycling processes.

Before the experiments were conducted, five 1000 mL volume glass containers were filled with 500 mL of investigated wastewater. Sorbent was added to four of the containers using a different quantity of sorbent in each sample. According to the highest porosity of natural sorbents, 1 g, 2 g, 3 g, or 4 g of straw or hydrophobic peat sorbent was used in each sample. Following experiments with mineral sorbent (high density compared with natural sorbents) were carried out using 5 g, 10 g, 20 g, 30 g, and 40 g respectively.

With the experimental equipment, these sorbents were mixed with the investigated wastewater by rotating at 60 r/min rate. The separation method is sedimentary process by which mineral sorbent falls to the bottom of sedimentation tank. This was carried out for the sedimentation time of 30 min between solids and liquids, before the water quality analysis. When hydrophobic peat and straw sorbents were added to water, the separation method was carried out in fluidized beds (30 min) where both sorbents were separated from the treated water on the top of used reservoirs. Both separation methods ensured that the samples taken for water quality analysis included the treated water only.

These samples were analyzed using the ICP emission instrument on Perkin–Elmer ICP-400 (The Perkin–Elmer Plasma 400 ICP Emission Spectrometer, PerkinElmer Inc., Waltham, MA, USA). All measurements investigated in this research were within the specified acceptance criterion of ±10% of the known value, and the typical deviation for most concentrations estimated less than 3%.

The quality of the water obtained was determined using indicators considered to be the most important: concentration of oil products, total suspended solids, total iron concentration, turbidity, color, conductivity, pH, and Chemical Oxygen Demand (COD). A portable conductivity measurement device (Cond315i, WTW, Weilheim, Germany) and pH-meter instrument (WTW pH 323, WTW, Weilheim, Germany) were used to measure individual samples of wastewater, conductivity, pH, and temperature in the tank. The infrared spectrophotometric analysis (IR) method was used to analyze the oil concentration. In the course of the experiment, a spectrophotometer (Spectroquant Nova 60 MERCK, Merck Group, Darmstadt, Germany) was used to measure turbidity and the color of wastewater (before and after sorption). Chemical Oxygen Demand (COD) is a chemical test, used as a chemical oxidation of the wastewater samples. The dichromate COD method measures close to the maximum possible oxygen demand of the wastewater sample using the standard method ISO 15705:2002—a specific version of the sealed tube (Micro) method [12]. Total suspended solids were measured after filtering of the obtained sample through a 0.45 µm filter membrane and all filters were dried at 105 °C and weighed. Iron concentrations in wastewater were determined using atomic absorption spectrometry at the laboratory and by colorimetric methods directly on site of wastewater treatment plants. The data were evaluated using “MathCad” created software with a type I error (a) of 0.05.

## 3. Results and Discussion

See Table 1 for wastewater quality data of the sample investigated from tank washing processes.

**Figure 1:**
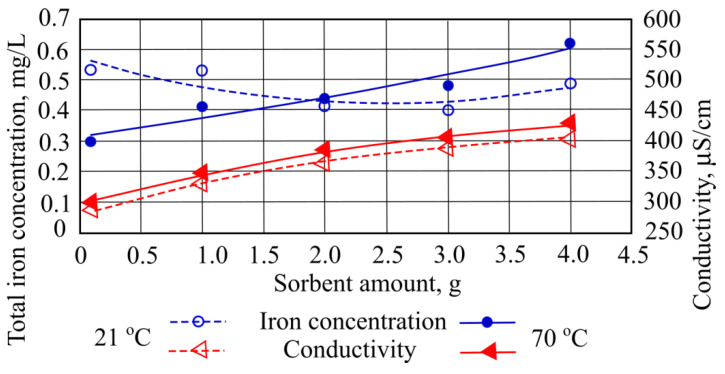
Total iron concentration and conductivity when different quantities of straw sorbent are added to the wastewater.

When 3 g of straw sorbent (the smallest amount) was added to the investigated wastewater at a temperature of 21 °C, the total iron concentration decreased to 0.4 mg/L. However, when the same amount is added to wastewater at a temperature of 70 °C, the pollution concentration increased (Figure 1).

**Figure 2:**
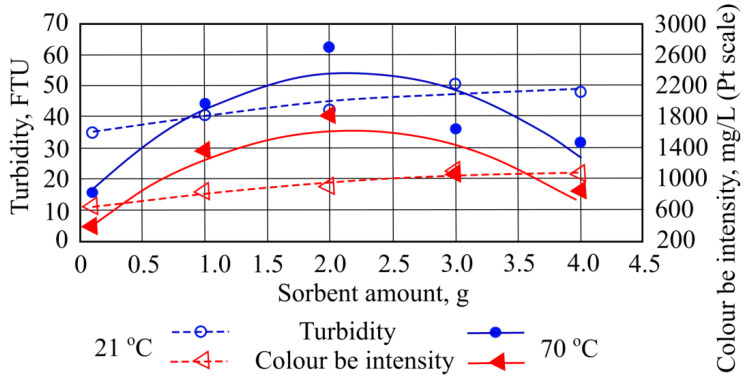
Turbidity values and color intensity when different quantities of straw sorbent are added to the wastewater.

When the wastewater was treated with 3 g of sorbent at 21 °C, the highest measured turbidity value 42.5 FTU was obtained. When the same amount of sorbent (3 g) is added to a water temperature of 70 °C, turbidity value decreases to 62.0 FTU (Figure 2).

Adding different quantities of straw sorbent to the wastewater at a temperature of 21 °C increases the color parameter from 659.02 mg/L to 1101.69 mg/L. However, when sorbent is added at 70 °C temperature, color increased to 1799.59 mg/L (Figure 2). Obtained total suspended solids interacted with used sorbents and were significantly affected by numerous factors. The wastewater from oil reservoirs washing processes was affected by rainfall and catchment runoff; reservoir corrosion; sediment disturbance, for example, by pumps; waste discharge; wastewater flow; and transported oil types. Under the pH conditions prevalent in wastewater treatment plants, iron salts are unstable and react with transported compounds to form insoluble hydroxides, which settle out as rust-colored silt. Wastewater in which this occurs can stain oil transportation reservoirs and plumbing fixtures. In the pumping system, iron compounds can settle out in the pipelines and gradually reduce the flow rate through the pipes.

**Figure 3:**
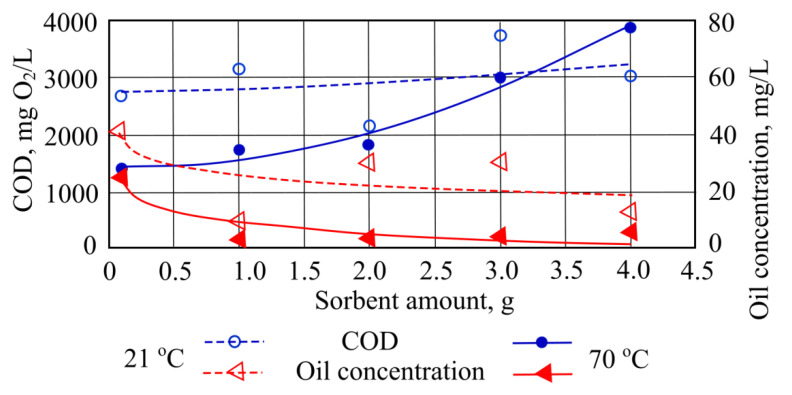
COD and oil concentrations when different quantities of straw sorbent are added to the wastewater.

When measuring COD at a water temperature of 21 °C using only 2 g of sorbent, most of the pollutants were removed (Figure 3). In other cases, this COD indicator was found to be higher than the initial value. When adding amounts of sorbent into 70 °C temperature water, the COD indicator increased until it reached its highest value of 3868.8 mgO_2_/L after adding 4 g of straw sorbent (Figure 3).

When straw sorbent was introduced to the investigated wastewater at the controlled temperatures, oil product concentration values decreased. The best result was obtained when the temperature of the wastewater was 21 °C and 1 g of straw sorbent was used. In this case the sorbent absorbed 33 mg of oil pollutant. The most effective oil product sorption at 70 °C occurs when the straw sorbent adsorbed 22.5 mg of oil products (Figure 3).

**Figure 4:**
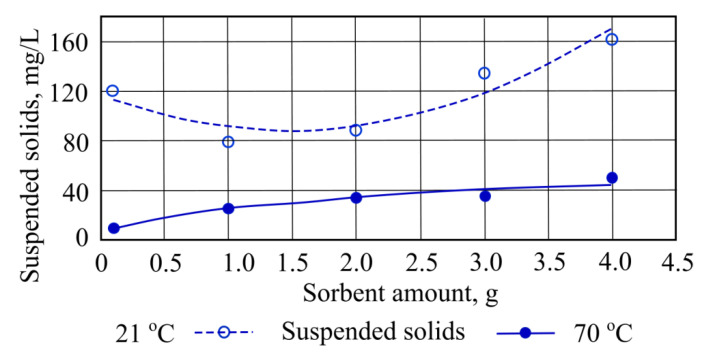
Suspended solids concentrations when straw sorbent is added to the wastewater.

It was found that a decrease in sorption capacity [13] occurs when suspended solids are measured at a wastewater temperature of 21 °C, and when straw sorbent is added in quantities of 1 g and 2 g, during which 41 mg/L of suspended solids was removed. However, adding a higher quantity of straw sorbent results some increases of the concentration of suspended solids from 42 mg/L to 161.8 mg/L (Figure 4).

**Figure 5:**
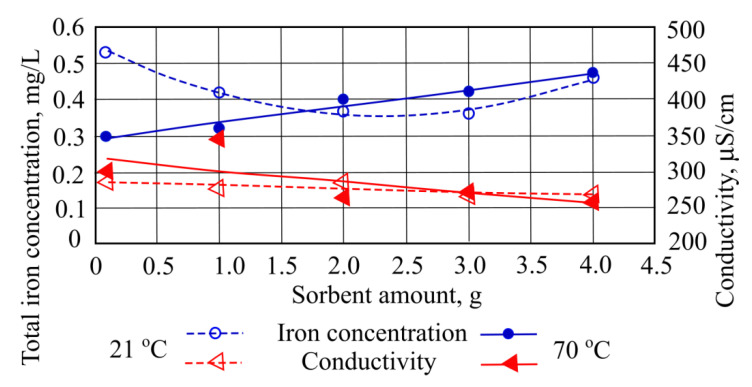
Total iron concentration and conductivity when different quantities of hydrophobic peat sorbent is added to the wastewater.

**Figure 6:**
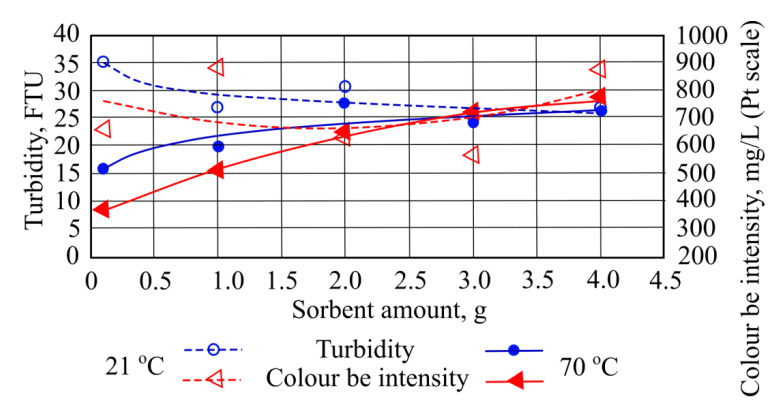
Turbidity and color be intensity values when different quantities of hydrophobic peat sorbent are added to the wastewater.

**Figure 7:**
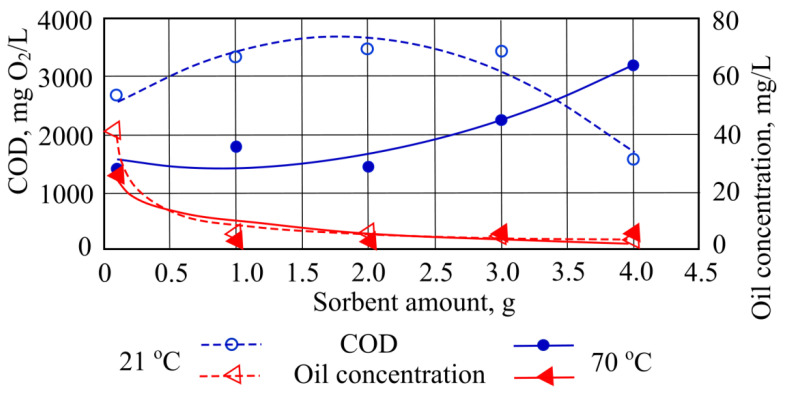
Chemical oxygen usage and oil concentration when different quantities of hydrophobic peat sorbent are added to the wastewater.

**Figure 8:**
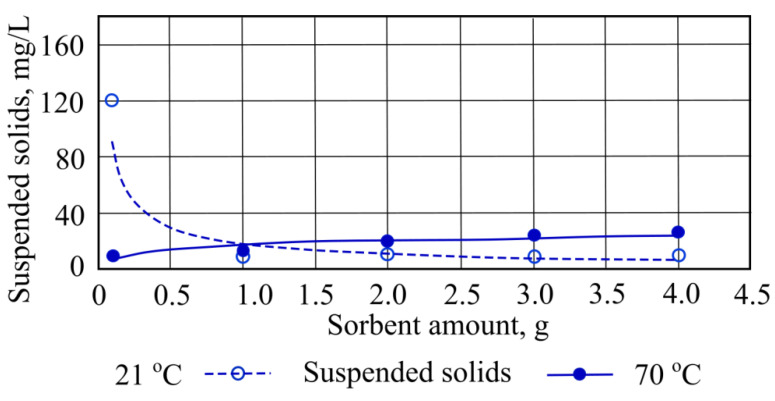
Suspended solids concentration when different quantities of hydrophobic peat sorbent are added to the wastewater.

Experimental results obtained from peat sorbent at a wastewater temperature 21 °C are presented below in Figure 5, Figure 6, Figure 7 and Figure 8.

The biggest reductions are noted in total iron concentration [14], by 0.17 mg/L from the starting value when the investigated water is at 21 °C and 3 g of hydrophobic peat sorbent has been added. When quantities of hydrophobic peat sorbent (4 g) are added at a water temperature of 70 °C, the total iron concentration captured is higher, and pollutant concentration is higher by 0.17 mg/L from the starting value (Figure 5). After adding the highest sorbent amount (4 g) into both temperatures of investigated waters, total iron concentration value was measured to be very similar—0.46–0.47 mg/L.

Figure 5 shows that in both temperatures (low and high) of the investigated water (and with the addition of hydrophobic peat sorbent), the indicator of conductivity falls at most by 21 μS/cm from initial value at 21 °C water, and by 38 μS/cm from initial value at 70 °C.

When measuring the turbidity indicator during the experiment using different investigated water temperatures, it was noted that at 21 °C water turbidity falls the most by 9.8 FTU from its initial value. However, at 70 °C when adding quantities of the sorbent, the turbidity indicator does not fall, while adding higher masses, its turbidity increases up to 11.8 FTU from a starting value (Figure 6).

Adding different hydrophobic peat sorbent masses into the water with a temperature of 21 °C produced inconsistent results. For example, by adding quantities of the smallest and highest sorbent masses (1 g and 4 g), color intensity indicator increases up to 218.34 mg/L (Pt scale) from the starting value. When 2 g and 3 g of this sorbent is added into water of the same temperature, color intensity falls to 96.71 mg/L (Pt scale) from the starting value. By adding different masses of hydrophobic peat sorbent to 70 °C temperature water, higher color intensity values were captured. The highest value (396.81 mg/L (Pt scale) rise from initial value) was determined when using 4 g of the sorbent (Figure 6).

The experiments found that chemical oxygen demand removal at 21 °C temperature occurs only when the highest mass of hydrophobic peat sorbent is used. In these conditions the decrease in chemical oxygen usage indicator was 1094.4 mg/L. In other conditions the concentration of this pollutant rises from 2668.8 mg/L to 3494.4 mg/L. When this indicator was investigated using water at a temperature of 70 °C, chemical oxygen usage sorption was recorded only when 2 g quantities of this sorbent were added. In other conditions the rise in pollutant was measured up to 1795.2 from the starting value (Figure 7).

Analysis of the results obtained where hydrophobic peat sorbent has been used showed that at both 21 °C and 70 °C the sorbent is highly efficient in adsorbing oil products. The best result is obtained by adding 4 g of sorbent into the water at 21 °C, and 2 g into water that is 70 °C. Water at room temperature (Figure 7) increases the capacity of sorbent sorption to remove oil products.

During experiments to investigate the concentration of suspended solids, we noted that at 70 °C the pollutant concentration is significantly lower when compared to water at room temperature. When adding different hydrophobic peat sorbent masses into 70 °C temperature water that contains a high suspended solids concentration, an increase is observed that reaches 18 mg/L at most from the starting concentration. When hydrophobic peat sorbents were added to water at 21 °C, there was a notable decrease in the concentration of suspended solids (Figure 8).

**Figure 9:**
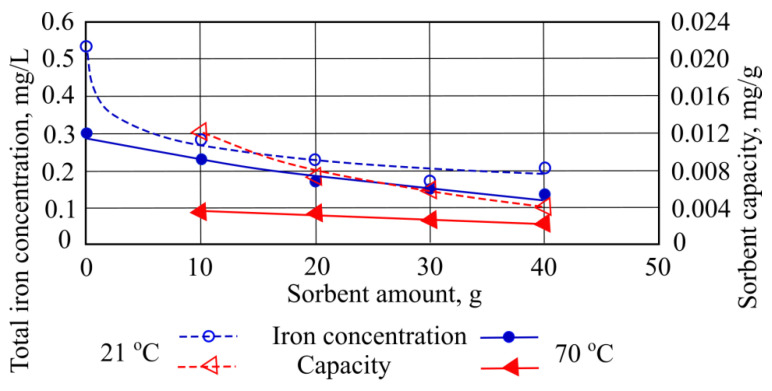
Total iron concentration and sorption capacity of mineral granulated sorbent.

Total iron is effectively adsorbed when granulated mineral sorbent granules were added in water at 21 °C and 70 °C. At 21 °C the most effective sorption takes place at 30 g of sorbent when 0.36 mg/L of total iron is adsorbed. At 70 °C the most effective sorption takes place when the highest quantities of sorbent are added, and during the sorption, the highest amount of pollutant is adsorbed (Figure 9).

Figure 9 shows the highest sorption capacity (0.0035 mg/g) differs only by a factor of 1.6 from the lowest measurement (0.002).

**Figure 10:**
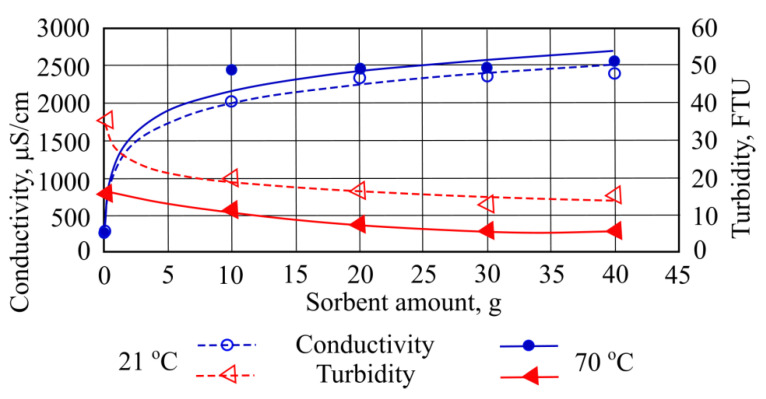
Electrical conductivity and turbidity using different quantities of mineral granulated sorbent.

Figure 10 shows results of electrical conductivity change when different quantities of mineral granulated sorbent were added. The highest values of this indicator in 21 °C and 70 °C water were obtained when the highest amount of sorbent was added. From a starting value they differ by 8.4–8.6 times.

When investigating different quantities of mineral granule sorbent and their influence on the water under investigation (i.e., measuring turbidity indicators), it was noted that at a room temperature of 21 °C the best results were obtained after adding 30 g of granulated sorbent. High turbidity removal from water was noted in hot water (70 °C) when adding larger quantities of mineral granule sorbent, so that lower turbidity indicator values are obtained. In this case, the highest turbidity removal efficiency was measured using 40 g of granulated sorbent (Figure 10).

**Figure 11:**
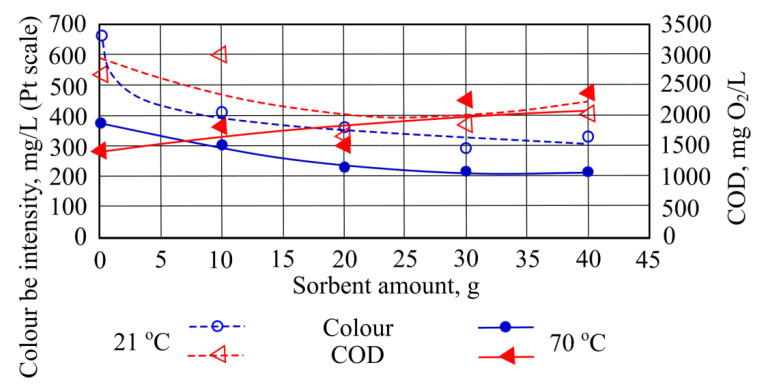
Color and chemical oxygen demand, when different quantities of mineral granulated sorbent are added to the water.

When adding different quantities of mineral granule sorbent to the investigated water, color intensity was notably decreased. Based on the results obtained, the more effective color removal takes place at room temperatures of the investigated water (Figure 11).

The best result is obtained by adding 20 g of mineral granulated sorbent, and in these conditions the removal efficiency of chemical oxygen demand is 37.4%. At the higher temperature, water chemical oxygen demand is not removed. When adding 30 g of mineral granule sorbent COD values rise by 835.2 mg/L from starting value (Figure 11).

**Figure 12:**
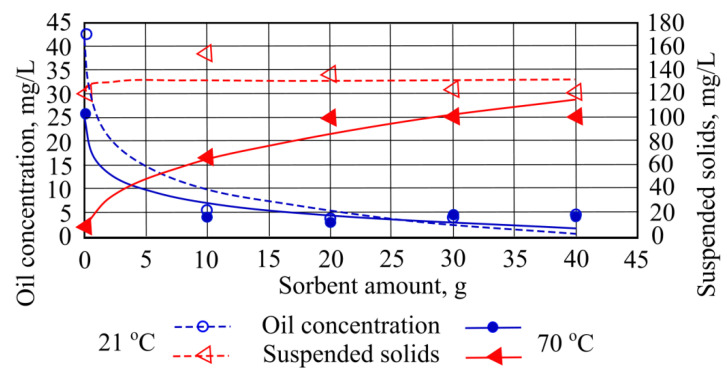
Oil concentration and suspended solids when different quantities of mineral granulated sorbent are added to the water.

When investigating the influence of granulated mineral sorbent on oil product concentration in different temperature water, it was found that in both cases oil product concentration decreased when the smallest quantity of mineral sorbent was added. (Figure 12).

When investigating the concentration of suspended solids at 70 °C when using different sorbent amounts, we noted that this indicator rises as the quantity of sorbent added is increased. However, when using 20 g, 30 g, and 40 g of sorbent masses, results changed insignificantly—only by 1 mg/L (Figure 12).

**Figure 13:**
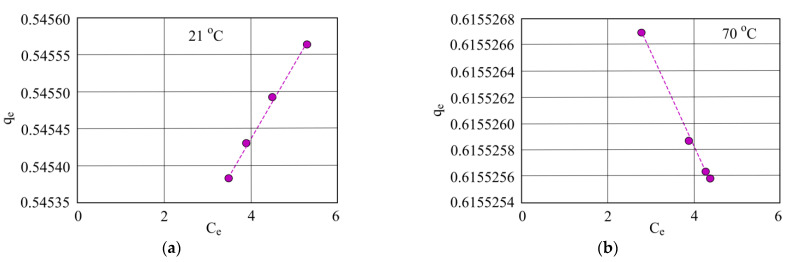
Freundlich isotherm when investigated water is at (**a**) 21 °C and (**b**) 70 °C using granulated mineral sorbent.

The results obtained were used to compose Freundlich isotherms. Figure 13 shows the results for granulated mineral sorbent. The Freundlich isotherms are at 21 °C (a) and 70 °C (b) of investigated water.

$$q_{s}=a_{F}C_{e}^{1/n},$$

Here:

$a_{F}$ and *n* are Freundlich constants;

$C_{e}$ is equilibrium adsorbed compounds concentration in water, mg/L.

Plotting Freundlich isotherm on a logarithmic scale, $a_{F}$ and *n* are Freundlich constants estimated from the intercept and the slope, accordingly. Using numerical values, *a_F_* expresses the adsorbent capacity; the higher its value, the larger the capacity; *n* is the heterogeneity constant. The more heterogeneous the sorbent, the closer *n* value is to zero. Freundlich isotherm gives more exact results than the Langmuir isotherm for a large selection of heterogeneous sorbents and complex sorption admixtures systems.

**Figure 14:**
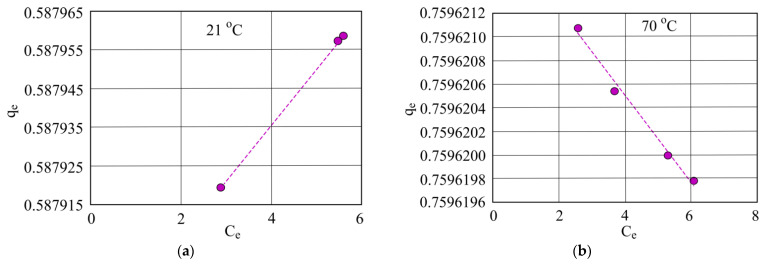
Freundlich isotherm when investigated water temperature is at (**a**) 21 °C and (**b**) 70 °C using hydrophobic peat sorbent.

The Freundlich isotherm shown above demonstrates that when investigated water is at 21 °C and oil concentration rises, sorbent sorption capacity decreases. However, at the higher temperature of 70 °C when oil product concentration is higher, sorbent sorption capacity falls (Figure 14).

In the Freundlich isotherms (Figure 14) adsorbing oil products by using hydrophobic peat sorbent, it was determined that oil concentration rises in the investigated water at room temperature, and sorption capacity also rises. This is consistent with the previously shown isotherms. For the investigated water at 70 °C it is anticipated that oil concentration will rise and the sorbent sorption capacity lower accordingly.

**Figure 15:**
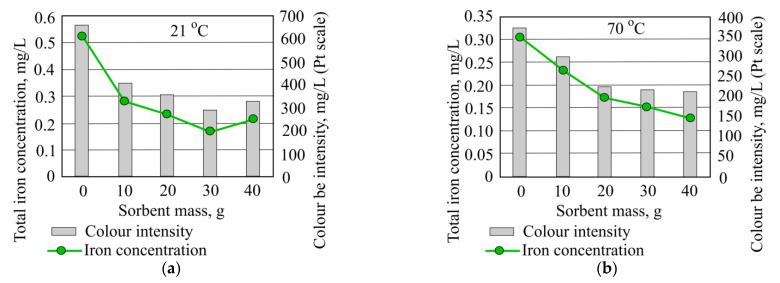
Total iron concentration and color intensity when investigated water is at (**a**) 21 °C and (**b**) 70 °C using granulated mineral sorbent.

When comparing the results of the experiment there is a noticeable connection between certain indicator results according to some references [15,16]. The linked indicator graph, Figure 15, shows that when color intensity value is at its highest, common iron concentration is also at its highest, while the color intensity value at its lowest matches with the lowest value of the common iron concentration. This trend is noticeable both at temperatures of 21 °C and at 70 °C.

**Figure 16:**
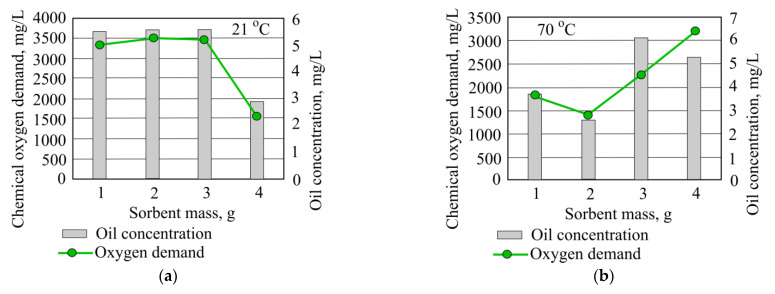
Chemical oxygen demand and oil concentration when investigated water is at (**a**) 21 °C and (**b**) 70 °C using hydrophobic peat sorbent.

It was also noted that when using hydrophobic peat sorbent for experiments with sufficiently high chemical oxygen demand values, higher measures of oil product concentration are found, while measuring a lower value the chemical oxygen demand is also determined as being lower (Figure 16).

**Figure 17:**
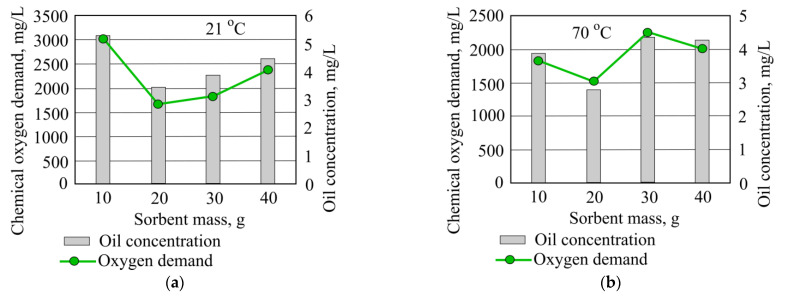
Chemical oxygen demand and oil concentration when investigated water is at (**a**) 21 °C and (**b**) 70 °C using granulated mineral sorbent.

The use of mineral granular sorbent for research produces a noticeable pattern (Figure 17) between chemical oxygen demand and oil concentration in water, similar to what is found when using hydrophobic peat sorbent.

The dosage effect is the capacity of selected sorbent, or the quantity required to remove an amount of targeted pollutant. The mechanism could be explained according to the total amount of investigated materials, e.g., for the biggest dosage, the highest obtained removal was 37 mg of oil per one gram of hydrophobic peat sorbent. In some cases, overdosing phenomena occurred with undesirable effects, i.e., increased concentrations values on the investigated materials. This was illustrated by increased turbidity values and color intensity when larger quantities of straw sorbent were added to the wastewater. The dosage effect on sorption performance or capacity is developed by equilibrium data of the model designed. Industrial technologists were evaluating color intensity and total iron concentration in preferably lowest values, thereafter the dosage effect was evaluated as efficient.

The authors of this article were looking for the most efficient sorbent and which mineral granulated sorbent is appropriate due to it being a wasted material from production sites, while building construction sectors are only interested in limited size fractions. Carbon based exhausted sorbents are very expensive to reuse or to manage in the environment [17,18]. Peat sorbent was investigated in the wastewater treatment plant of oil reservoir washing pollutant removal and it only provided some useful results. Some researchers were working with oil polluted water treatment from a related recycling and reuse of car wash water [19,20,21] and were interested in the efficient sorbent usage and they continue research with wasted material and similar sorbents. According to the Environmental Law of European Union we proposed to incorporate exhausted mineral sorbent in cement production materials for local market use.

The influence of the concentration on oil retention can be explained by the phenomenon of concentration polarization [22]. Generally, chemical sorption and catalytic processes allow us to have partial and selective removal in addition to an important production flow, which makes the sorption technique competitive when compared to other processes [23,24].

The combination of filtration technique with sorption process can lead us to an optimal treatment of oil and other pollutants from the reused water of oily reservoirs washing processes, with a lower cost than when using synthetic sorbents and without harming the environment [25,26]. Whereas many agricultural residue-derived sorbents have been reported [26,27], the breakthrough was explored by using porous Fe/C bio-char adsorbent and a novel phenolic foam-derived magnetic carbon foam.

## 4. Conclusions

1. The investigated sorbents can be used for the treatment of target wastewater. The selection of sorbents handling method will depend on the quantity of sorbents to be obtained from local sources: straw sorbent seasonal availability, peat excavating frequency, and wasted mineral sorbent accumulating at the cement production factory. Depending on the nature of sorbents, however, after the oily wastewater treatment, regenerating or additional methods of disposal may be necessary. The promising results were obtained with the mineral sorbent and future research will be focused on batch-type processes.
2. At 70 °C, the sorption capability of iron in the investigated water using mineral sorbent was reduced by 2.5 times when compared to water at 21 °C.
3. Raising the temperature of the water from 21 °C to 70 °C, lowers the effectiveness of all pollutant removal under investigation by 2.0–15.0% on average.
4. Removing total iron from water weakens the color of the investigated water due to trivalent iron ions, giving the water a pink or brown tinge.
5. The effect of the characteristics of the materials on the adsorption capacity was evaluated using removed amount of oil per one gram of every sorbent. Straw sorbent oil adsorption capacity was up to 33 mg/g; peat sorbent 37 mg/g, and mineral sorbent 1.83 mg/g.

## Figures and Tables

**Table 1:** The wastewater quality data of investigated samples from tank washing processes.

Nr.	Parameter	Measuring Unit	Value
1.	Total suspended solids	mg/L	119.80
2.	Total iron concentration	mg/L	0.53
3.	Turbidity	FTU	35.00
4.	Color	mL/L (Pt scale)	659.02
5.	Conductivity	μS/cm	287
6.	pH	pH	7.32
7.	COD	mg/L	1370.0
8.	Oil concentration	mg/L	42.00

## Data Availability

The data presented in this study are available on request from the corresponding author.
